# Siblings, shopping, and sustainability: Birth-order differences in green consumption

**DOI:** 10.3389/fpsyg.2023.1105072

**Published:** 2023-03-03

**Authors:** Tobias Otterbring, Christine Sundgot-Borgen, Solfrid Bratland-Sanda, Lise Katrine Jepsen Trangsrud

**Affiliations:** ^1^Department of Management, University of Agder, Kristiansand, Norway; ^2^Regional Department for Eating Disorders, Division of Mental Health and Addiction, Oslo University Hospital, Oslo, Norway; ^3^Department of Sport, Physical Education and Outdoor Studies, University of South-Eastern Norway, Kongsberg, Norway; ^4^Department of Health, Social and Welfare Studies, University of South-Eastern Norway, Kongsberg, Norway

**Keywords:** firstborns, laterborns, siblings, sustainability, environmental protection, pro-environmental consumption, green consumption, prosocial behavior

## Abstract

Several studies have examined the role of birth order in shaping human personality, but fewer have tested this variable in relation to other pressing issues. We conducted a birth-order study on green consumption, which enabled us to detect a small-to-moderate effect size equivalent to *r* = 0.15 or *d* = 0.30 with sufficient statistical power (*N* = 335). To capture green consumption, participants indicated their tendency to express the value of environmental protection through purchases and consumption behaviors. Firstborns (vs. laterborns) consistently expressed lower concerns linked to environmental protection in their purchase patterns. While the effect size of this finding was small-to-moderate by conventional standards and in direct contrast to the findings from a recent article on the same topic, these results could still be informative to address challenges associated with climate change considering the number of individuals with siblings in the world and the ease with which birth-order data can be collected.

## 1. Introduction

Francis Galton (1874) was among the first to highlight the role of birth order on academic achievements and personality. He believed that the oldest sons benefitted from the most stimulating intellectual environment and hence should be more likely than their siblings to end up as authoritative academics. More recent research suggests that he, at least partially, may have been right, but that the effect sizes of birth-order findings on cognitive abilities are small, with firstborns only slightly more intelligent than laterborns (Rohrer et al., 2015).

Despite multiple studies on the role of birth order in shaping human personality (for a meta-analysis, see Sulloway, 1995), the existing literature has largely neglected this variable in relation to other important aspects of social life (Salmon et al., 2016). Importantly, few studies have examined whether birth order can predict consumption-related outcomes, although some notable exceptions exist. For example, Saad et al. (2005) demonstrated that firstborns (vs. laterborns) were more susceptible to normative interpersonal influence in their purchase patterns and hence more inclined to comply with certain norms in their consumption responses (e.g., purchasing brands that others will approve of). In other words, laterborns were more likely to violate such norms, supporting former research which has discussed a later birth order in relation to more nonconforming characteristics (Sulloway, 1995). Similarly, Zemanek et al. (2000) found firstborns (vs. laterborns) to exhibit lower levels of materialism; Nancarrow et al. (1999) documented firstborns (vs. laterborns) to express a higher desire to talk to others before and/or after a high involvement purchase; and Berekson (1972) presented some evidence for the notion that firstborns (vs. laterborns) would be more prone to purchase life insurance.

Given the urgent need to tackle climate change (Stern, 2015; Campbell et al., 2018; Folwarczny and Otterbring, 2021), some studies have also linked birth order to a set of prosocial responses, which can be conceptualized as actions and attitudes that have other-oriented rather than self-centered benefits, including sharing, giving, helping, and cooperating for the greater good (Batson and Powell, 2003; Otterbring et al., 2021b; Pfattheicher et al., 2022). Engaging in green consumption is one example of prosociality, as purchasing sustainable products rather than conventional alternatives may mitigate climate change issues and benefit the planet (Gidlöf et al., 2021; Loebnitz et al., 2022). Accordingly, this Brief Research Report aimed to add to this stream of literature by examining whether firstborns and laterborns differ in prosocial responses linked to green consumption, hereinafter defined as the propensity to display values linked to environmental protection by means of one’s purchase patterns and the way products and services are consumed (Haws et al., 2014; Grønhøj and Hubert, 2022). This main objective is warranted not only due to the societal relevance of studying potential predictors of green consumption, but also because the birth-order literature is mixed with respect to whether firstborns or laterborns can be expected to exhibit the most prosocial responses in this consumption domain, as further delineated below.

## 2. Mixed messages in the birth-order literature

Otterbring and Folwarczny (2022) predicted and found that firstborns should be more prone than laterborns to engage in prosocial behavior linked to green consumption, with such pro-environmental responses recently portrayed as a prosocial kin care action (Palomo-Vélez and van Vugt, 2021). Considering that firstborns share parental responsibility in terms of caring for and teaching their younger siblings (Sulloway, 2001; Hughes et al., 2018), it seems reasonable that they should be more inclined to engage in green consumption. Further support for this thesis stems from findings that have linked birth order to both intelligence and conscientiousness, with firstborns being somewhat more intelligent and conscientious than laterborns (Sulloway, 1995; Rohrer et al., 2015). Therefore, as firstborns not only act as surrogate parents for their younger siblings (Pollet and Nettle, 2007; Su et al., 2014) but also tend to have a personality profile that is linked to responsibility-related aspects (Sulloway, 1995), it is plausible that they are more inclined than laterborns to engage green consumption, considering that such sustainable shopping responses are often referred to as a way of demonstrating responsibility (Wu and Yang, 2018; Yue et al., 2020). Following this line of logic, we formulate the following research question (RQ):

RQ1a: Are there birth-order differences in green consumption and, if so, are firstborns more inclined to engage in green consumption than laterborns?

However, other birth-order findings suggest the opposite outcome, such that laterborns may be more prone to participate in green consumption and similar acts of prosociality. Indeed, several studies suggest that laterborns are more prosocial than firstborns (Salmon et al., 2016; Okada et al., 2021), while simultaneously being warmer and more empathetic, less narcissistic, and more cooperative, altruistic, and other-oriented in their achievement goals (Paulhus et al., 1999; Eckstein et al., 2010; Carette et al., 2011; Prime et al., 2017). For example, using a large-scale population-based cohort dataset of more than 3,000 adolescents, Okada et al. (2021) recently found laterborns to score higher than firstborns on various prosociality measures, as captured through scores on the Strengths and Difficulties Questionnaire (SDQ; Goodman, 1997). Similarly, Salmon et al. (2016) found a moderate association between birth order and prosociality, such that laterborns displayed greater prosocial responses than firstborns on aspects such as empathy, social altruism, and family support. Furthermore, research on cooperative behaviors has revealed that laterborns generally tend to reciprocate more than firstborns in economic games, with birth order being a better predictor of such cooperativeness than factors such as age, gender, and income (Courtiol et al., 2009). Accordingly, as an alternative to RQ1a, we also acknowledge the contrasting possibility:

RQ1b: Are there birth-order differences in green consumption and, if so, are laterborns more inclined to engage in green consumption than firstborns?

## 3. Methods

### 3.1. Participants, exclusion criteria, and statistical power

The study was part of a larger, multi-national project, which examined people’s body image in nature across cultures (for further details, see Swami et al., 2022). The overall research project received ethical approval from the School Research Ethics Committee at Anglia Ruskin University (approval code: PSY-S19-015). For the purpose of the current study, the data were collected solely in Norway, were approved by the Norwegian Data Protection Service (ID 833522), and originally included a community sample comprising 360 participants. All participants provided written informed consent before getting access to the survey and were not compensated for taking part in the study. As far as can be ascertained, birth-order data were only collected in Norway to address the unique aim of the current research. Another portion of the Norwegian data, which focused on the relationship between physical activity arenas and adults’ body appreciation, appears in Sundgot-Borgen et al. (2022).

Given that prior research has found birth-order effects to be sensitive to participants’ ethnic origin even in neighboring countries (Saarela et al., 2016; Saarela and Kolk, 2021), we excluded participants who either described their ethnic affiliation as representing an ethnic minority (*n* = 16) or who were not sure about their ethnic affiliation (*n* = 7).^1^ Further, we excluded participants whose stated gender (“other”) was too infrequently represented for meaningful analyses (*n* = 2), leaving a final sample of 335 participants (76.72% female; *M*_age_ = 41.61, *SD* = 11.77) representing the ethnic majority in Norway. This sample size has a statistical power of approximately 80% to detect small-to-moderate effect sizes corresponding to *r* = 0.15 or *d* = 0.30, assuming a conventional alpha level of *α* = 0.05 (Cohen, 2013). Moreover, our sample size is larger than the sample sizes used in most former birth-order studies on consumption-related outcomes, which have typically included around 80 to 310 participants (e.g., Claxton, 1995; Claxton et al., 1995; Zemanek et al., 2000; Saad et al., 2005; Berisha et al., 2022).

### 3.2. Procedure and measures

Participants were recruited through university networks and social media (Facebook, Instagram, Twitter), and represented a heterogeneous sample from different regions of Norway. Of the entire sample, approximately 20% lived in the capital city or its suburbs (*n* = 66; 19.7%), with a similar share of participants living in provincial cities with more than 100,000 residents (*n* = 61; 18.2%) or in the rural areas of the country (*n* = 74; 22.1%). The largest proportion of participants lived in provincial towns with more than 10,000 residents (*n* = 134; 40.0%). The sample was relatively highly educated, with most participants indicating having either a postgraduate degree (*n* = 160; 47.8%) or an undergraduate degree (*n* = 118; 35.2%). Less than 1% of participants indicated having no formal education (*n* = 2; 0.6%) or only primary education (*n* = 1; 0.3%). In terms of marital status, most participants were either married (*n* = 136; 40.6%) or in a committed relationship (*n* = 124; 37.0%), although some were single (*n* = 68; 20.3%) or indicated “other” as their marital status (*n* = 7; 2.1%).

Participants filled out a series of measures through an online link, including the well-validated 6-item GREEN scale (Haws et al., 2014), which contains items such as “My purchase habits are affected by my concern for our environment” (see the Appendix for all scale items). Responses were given on a 7-point scale (1 = strongly disagree; 7 = strongly agree) and were averaged to form a composite GREEN consumption index (α = 0.91). Given that birth-order effects have been widely discussed in relation to personality traits (Sulloway, 1995; Eckstein et al., 2010; Rohrer et al., 2015), participants further replied to the five-item personality inventory (FIPI), which captures each of the Big Five personality traits with a single-item measure per trait (Gosling et al., 2003). Participants indicated their responses on a 5-point scale for the personality items (1 = strongly disagree; 5 = strongly agree). As common method bias can be effectively mitigated by minimizing common scale properties (Podsakoff et al., 2003; Elbæk et al., 2022; Gasiorowska et al., 2022), the fact that we used different response alternatives across the survey (i.e., 5- and 7-point scales combined with categorical questions) means that this bias form should have been reduced (Podsakoff et al., 2012; Mellewigt et al., 2018; Otterbring et al., 2021a). We found no general birth-order differences on any of the Big Five personality traits (all *p*s > 0.16) and these traits will not be discussed further unless explicitly stated.

Data on participants’ sibship size, gender, age, and “only-child” status were collected to account for these variables in the analyses. Mirroring previous birth-order research (Rohrer et al., 2015; Otterbring and Folwarczny, 2022), we did not distinguish between participants who had full, half, step, or adoptive siblings. Regarding sibship size, however, there are more laterborns in larger sibships, and differences between firstborns and laterborns may thus occur due to laterborns being more likely to be born into families with a lower socioeconomic status, which can be associated with other individual differences (Rohrer et al., 2015; Otterbring and Folwarczny, 2022). To explicitly account for this potential bias source, participants indicated their financial security by replying to the following question: “Compared to others of your own age in your country, how financially secure do you feel?” Responses were provided through the categories: “less secure” (*n* = 44; 13.1%), “same” (*n* = 188; 56.1%), and “more secure” (*n* = 103; 30.7%). However, controlling for financial security in our analyses did not change the nature and significance of our results, and there was no significant correlation between sibship size and financial security (*r* = −0.06, *p* = 0.290) or between birth order and financial security (*r* = 0.03, *p* = 0.586), thus ruling out financial security as a crucial confound.

## 4. Results

We report our analyses in the following order: First, we conduct an independent samples *t*-test to examine whether firstborns (*n* = 167) differ from laterborns (*n* = 168) on the GREEN scale. Next, we present the partial correlation between birth order and green consumption while controlling for participants’ age, gender, sibship size, and “only-child” status. Subsequently, we report the results of a multiple linear regression, using birth order, age, gender, sibship size, and “only-child” status as the predictors, and the GREEN scale as the outcome variable. Finally, to show robustness of our findings, we test for birth-order differences between participants whose birth order equals first (*n* = 167), second (*n* = 101), or third (*n* = 48), accounting for 94.33% (*N* = 316) of the sample used in our main analyses. Following Otterbring and Folwarczny (2022), who excluded categories that made up less than 10% of the sample due to small cell sizes and hence insufficient statistical power to detect modest effects for these categories, we omit birth orders of 4 and higher (5.7%) in these final robustness tests; see Table 1 for the birth order and sibship size distributions in our sample and Table 2 for the zero-order correlations between our focal variables.

**Table 1:** Birth order and sibship size percentages.

Birth order	Percent	Cumulative percent	Sibship size	Percent	Cumulative percent
1	49.9	49.9	1	7.5	7.5
2	30.1	80.0	2	37.6	45.1
3	14.3	94.3	3	36.4	81.5
4	3.9	98.2	4	11.3	92.8
≥5	1.8	100.0	≥5	7.2	100.0

The highest birth order was 12, and the largest sibship size was 15 in the present study. Firstborns include “only-child” participants (*n* = 25; 7.5%).

**Table 2:** Grand means (with SDs) or probabilities of key variables and their zero-order correlations.

	*M (SD)* or %	1	2	3	4	5
Birth order (1 = first; 0 = later)	49.85% first					
Age in years (continuous)	41.61 (11.77)	−0.01				
Gender (1 = male; 0 = female)	23.28% male	−0.06	0.16**			
Sibship size (continuous)	2.80 (1.31)	−0.26***	0.13*	0.07		
Only child (1 = yes; 0 = no)	7.46% only child	0.19***	0.04	−0.08	−0.39***	
Green consumption (1–7)	5.08 (1.14)	−0.14**	0.20***	0.04	0.08	−0.04

*p ≤ 0.05; **p ≤ 0.01; ***p ≤ 0.001.

### 4.1. Independent samples *t*-test

Firstborns (*M*_first_ = 4.92, *SD* = 1.16) were found to score significantly lower than laterborns (*M*_later_ = 5.23, *SD* = 1.11) on the GREEN scale (*t*(333) = −2.51, *p* = 0.012, *d* = −0.28, 95% CI of *d* = [−0.49, −0.06]), indicating that their purchases and consumption preferences reflect lower concerns for environmental protection^2^. These findings go directly against RQ1a but support the competing possibility, as postulated by RQ1b.

### 4.2. Partial correlation

The partial correlation between birth order and the GREEN scale remained significant even after controlling for participants’ age, gender, sibship size, and “only-child” status (*r*_partial_ = −0.13, 95% CI of *r*_partial_ = [−0.23, −0.02], *p* = 0.022), again leaving RQ1a unsupported while offering additional support for RQ1b.

### 4.3. Multiple linear regression

Within the context of birth-order effects, the GREEN scale yielded a significant overall model (*F*(5, 329) = 4.07, *p* = 0.001, *R*^2^ = 0.06), with birth order (*b* = −0.29, 95% CI of *b* = [−0.54, −0.04], *β*_standardized_ = −0.13; *p* = 0.022) and age (*b* = 0.02, 95% CI of *b* = [0.01, 0.03], *β*_standardized_ = 0.20; *p* < 0.001) as significant predictors, and with participants’ gender (*b* = −0.02, 95% CI of *b* = [−0.31, 0.27], *β*_standardized_ = −0.01; *p* = 0.904), sibship size (*b* = 0.01, 95% CI of *b* = [−0.09, 0.12], *β*_standardized_ = 0.02; *p* = 0.803), and “only-child” status (*b* = −0.08, 95% CI of *b* = [−0.58, 0.42], *β*_standardized_ = −0.02; *p* = 0.754) as nonsignificant predictors. Thus, laterborns (vs. firsborns) as well as older (vs. younger) participants scored higher on the GREEN scale, whereas all other predictors were unassociated with the scores on this scale. Together, these results yet again leave RQ1a unsupported but provide converging evidence for RQ1b.

### 4.4. Robustness checks

A one-way ANOVA found a significant impact of birth order (first, second, third) on the GREEN scale (*F*(2, 313) = 3.42, *p* = 0.034, $\eta_{p}^{2}$ = 0.02). Follow-up planned contrasts revealed that firstborns (*M*_first_ = 4.92, *SD* = 1.16) scored significantly lower than participants whose birth order equaled second and third (*t*(313) = −2.48, *p* = 0.014, *d* = −0.58, 95% CI of *d* = [−1.04, −0.12]), whereas these latter groups did not differ significantly (*M*_second_ = 5.10, *SD* = 1.12 vs. *M*_third_ = 5.40, *SD* = 1.06; *t*(313) = −1.46, *p* = 0.145, *d* = −0.26, 95% CI of *d* = [−0.60, 0.09]). As in all former analyses, these findings go against RQ1a but add further robustness to RQ1b. Controlling for participants’ age, gender, sibship size, and “only-child” status did not change the nature or significance of these results (*F*(2, 309) = 3.21, *p* = 0.042, $\eta_{p}^{2}$ = 0.02).

## 5. Discussion

The current study sought to address the mixed findings pertaining to the link between birth order and prosociality in the domain of green consumption. In direct contrast to the findings from a recent article (Otterbring and Folwarczny, 2022), but consistent with a broader stream of literature on birth-order effects on aspects such as prosocial behavior, cooperation, and altruism (e.g., Eckstein et al., 2010; Salmon et al., 2016; Prime et al., 2017; Okada et al., 2021), we found robust evidence for the notion that laterborns (vs. firstborns) were more prone to purchase and consume products and services sustainably.

Our obtained effect size (*d* = −0.28) lies between the 30th and 35th percentile compared to the magnitude of published effect sizes in personality and social psychology (Gignac and Szodorai, 2016; see also Götz et al., 2022) and is similar in strength to the link between self-disclosure and likability (Meyer et al., 2001). Yet, this finding may still be ultimately consequential considering the number of people with siblings in the world and the ease with which birth-order measures can be collected (Funder and Ozer, 2019; Otterbring and Festila, 2021).

The present work is strengthened by a well-validated instrument (i.e., the GREEN scale) and multiple control variables, which jointly increase internal validity. However, selection bias constitutes a potential confound, as participants who voluntarily took part in the study—advertised as focusing on nature exposure and well-being—can be assumed to be particularly interested in nature and, by extension, sustainable actions.

The data at hand are based on citizens aged 18–83 years from a wide range of different urban and rural areas across Norway. This should make our data source more representative in terms of age and regional residence compared to the typical samples used in the academic literature. Indeed, scholars typically restrict themselves to convenience samples of WEIRD individuals (i.e., data collected in Western, educated, industrialized, rich, and democratic societies), normally in the form of university students or paid online panel participants (Masuda et al., 2020; Muthukrishna et al., 2020; Eguren et al., 2021; Otterbring, 2021). Nevertheless, considering that the current research was based on a community sample, we cannot automatically infer that our data are representative at the population level. In fact, our sample may well be less representative than a typical student sample on certain aspects. Therefore, future research may benefit from reliance on representative samples, and should preferably be conducted in other cultural contexts with different sample types to test the generalizability of our obtained results. Optimally, such future scholarly work should also measure and control for further confounding factors that may influence sibling dynamics (Keller et al., 2015; Grønhøj, 2016; Wu et al., 2018; Halder et al., 2020; Hou et al., 2020).

Otterbring and Folwarczny (2022) used the same GREEN scale as in the present investigation on a sample of online panel participants who had English as their first language and found firstborns (vs. laterborns) to show more pro-environmental consumption values, while the current research revealed the reverse among a community sample of Norwegian participants. Thus, there may be some constraints on generality pertaining to birth-order effects (Van Bavel et al., 2016; Simons et al., 2017; Kerr et al., 2018; Otterbring et al., 2022).

Interestingly, although Otterbring and Folwarczny (2022) failed to find a general link between birth order and susceptibility to normative interpersonal influence, their sub-group analyses based on younger consumers found that these participants were more susceptible to such influence if they were firstborns (vs. laterborns). In other words, laterborns were more inclined to violate certain social norms compared to firstborns, consistent with the notion that laterborns have a more rebellious disposition, characterized by nonconformism and innovativeness (Sulloway, 1995; Saad et al., 2005). Similarly, by restricting the current sample to participants aged 18–40 years (*N* = 162; 48.4% of the total sample), we largely replicated the nature and effect size of the birth-order difference in green consumption (*M*_first_ = 4.72, *SD* = 1.25 vs. *M*_later_ = 5.06, *SD* = 1.08; *t*(160) = −1.88, *p* = 0.062, *d* = −0.30), but also found that laterborns in this age group scored significantly higher than firstborns on the Big Five personality trait of Openness (*M*_first_ = 3.97, *SD* = 0.88 vs. *M*_later_ = 4.25, *SD* = 0.82; *t*(160) = −2.06, *p* = 0.041, *d* = −0.32), with this trait also correlating significantly with green consumption (*r* = 0.18, *p* = 0.021). As such, it is possible that our birth-order findings on green consumption, at least among participants of younger ages, can be attributed to trait differences in openness. Because the consumption practices that are adopted in early adulthood typically follow a habitual pattern (Machín et al., 2020; Ragelienė and Grønhøj, 2020; Perkovic et al., 2022), this may explain our persistent birth-order effect in green consumption across ages, as documented in our main analyses, despite that the trait difference in openness between firstborns and laterborns vanished over time in our study and hence did not apply to participants aged 41–83 years. Future research should try to test this possibility.

Another fruitful avenue for future research is to more explicitly examine whether green consumption is just one of many possible ways to exhibit prosociality or, alternatively, whether this consumption form differs on important dimensions from broader measures of prosociality or altruism. Although previous research has found a moderate association between general prosocial attitudes and green consumption (do Paço et al., 2019), this also means that birth-order effects on one of these constructs do not necessarily predict responses on the other. Accordingly, as our survey did not contain any generic measures of prosocial tendencies, further studies may benefit from simultaneously testing for birth-order differences on more than one facet of prosociality. Moreover, there is variability both between and within cultures regarding political values (e.g., liberal vs. conservative), and such values often include opinions about green consumption (Elliott, 2013; Gustafson et al., 2019; Bravo and Farjam, 2022), suggesting that participants’ political preferences may be important to control for in academic work on sustainability-oriented aspects.

Parents’ sustainable consumption (e.g., buying eco-labeled products) and the way they communicate about environmentally friendly aspects (e.g., sorting household waste) are important factors in shaping children’s pro-environmental responses (Grønhøj and Thøgersen, 2017; Gong et al., 2022). It is possible that parents develop more pro-environmental values when they get a larger number of children, as they may increasingly consider their children’s future under such circumstances. If so, these values should be more immediately transferred to laterborns relative to firstborns, influencing them from an earlier age (*cf.* Grønhøj, 2006; Grønhøj and Thøgersen, 2009). In contrast, firstborns may be more prone to stay loyal to their parents’ initial values and may therefore exhibit less sustainable attitudes and behaviors. Such an interpretation would explain why laterborns scored higher than firstborn on the GREEN scale in the current investigation and hence calls for further empirical research.

## Data availability statement

The raw data supporting the conclusions of this article will be made available by the authors, without undue reservation.

## Ethics statement

The overall research project received ethical approval from the School Research Ethics Committee at Anglia Ruskin University (approval code: PSY-S19-015). For the purpose of the current study, the data were collected solely in Norway, and the project was approved by the Norwegian Data Protection Service (ID 833522). The patients/participants provided their written informed consent to participate in this study.

## Author contributions

TO: conceptualization, methodology, investigation, formal analysis, writing – original draft, and writing – review and editing. CS-B: methodology, investigation, and writing – review and editing. SB-S: methodology, investigation, data curation, writing – review and editing. LT: methodology, investigation, and writing – review and editing. All authors contributed to the article and approved the submitted version.

## Conflict of interest

The authors declare that the research was conducted in the absence of any commercial or financial relationships that could be construed as a potential conflict of interest.

## Publisher’s note

All claims expressed in this article are solely those of the authors and do not necessarily represent those of their affiliated organizations, or those of the publisher, the editors and the reviewers. Any product that may be evaluated in this article, or claim that may be made by its manufacturer, is not guaranteed or endorsed by the publisher.

## Footnotes

^1^Controlling for ethnicity in our main analyses instead of excluding participants based on their ethnic affiliation did not change the nature or significance of our results.

^2^The size of our *negative* link between birth order and green consumption is significantly different from the *positive* link reported by Otterbring and Folwarczny (2022), *Z* = −3.69, *p* < 0.001.

## 6. Appendix: Scale items, green consumption

The GREEN Scale (Haws et al., 2014; 1 = strongly disagree; 7 = strongly agree; α = 0.91):

1. It is important to me that the products I use do not harm the environment.
2. I consider the potential environmental impact of my actions when making many of my decisions.
3. My purchase habits are affected by my concern for our environment.
4. I am concerned about wasting the resources of our planet.
5. I would describe myself as environmentally responsible.
6. I am willing to be inconvenienced in order to take actions that are more environmentally friendly.
